# Accurate and fast detection of tomatoes based on improved YOLOv5s in natural environments

**DOI:** 10.3389/fpls.2023.1292766

**Published:** 2024-01-11

**Authors:** Philippe Lyonel Touko Mbouembe, Guoxu Liu, Sungkyung Park, Jae Ho Kim

**Affiliations:** ^1^ Department of Electronics Engineering, Pusan National University, Busan, Republic of Korea; ^2^ School of Computer Engineering, Weifang University, Weifang, China; ^3^ R&D Center, Univalsoft Joint Stock Co., Ltd., Shouguang, China; ^4^ Exsolit Research Center, Yangsan, Republic of Korea

**Keywords:** artificial intelligence, tomato detection, attention mechanism, BiFPN, YOLOv5, computer vision, agriculture

## Abstract

Uneven illumination, obstruction of leaves or branches, and the overlapping of fruit significantly affect the accuracy of tomato detection by automated harvesting robots in natural environments. In this study, a proficient and accurate algorithm for tomato detection, called SBCS-YOLOv5s, is proposed to address this practical challenge. SBCS-YOLOv5s integrates the SE, BiFPN, CARAFE and Soft-NMS modules into YOLOv5s to enhance the feature expression ability of the model. First, the SE attention module and the C3 module were combined to form the C3SE module, replacing the original C3 module within the YOLOv5s backbone architecture. The SE attention module relies on modeling channel-wise relationships and adaptive re-calibration of feature maps to capture important information, which helps improve feature extraction of the model. Moreover, the SE module’s ability to adaptively re-calibrate features can improve the model’s robustness to variations in environmental conditions. Next, the conventional PANet multi-scale feature fusion network was replaced with an efficient, weighted Bi-directional Feature Pyramid Network (BiFPN). This adaptation aids the model in determining useful weights for the comprehensive fusion of high-level and bottom-level features. Third, the regular up-sampling operator is replaced by the Content Aware Reassembly of Features (CARAFE) within the neck network. This implementation produces a better feature map that encompasses greater semantic information. In addition, CARAFE’s ability to enhance spatial detail helps the model discriminate between closely spaced fruits, especially for tomatoes that overlap heavily, potentially reducing the number of merging detections. Finally, for heightened identification of occluded and overlapped fruits, the conventional Non-Maximum-Suppression (NMS) algorithm was substituted with the Soft-NMS algorithm. Since Soft-NMS adopts a continuous weighting scheme, it is more adaptable to varying object sizes, improving the handling of small or large fruits in the image. Remarkably, this is carried out without introducing changes to the computational complexity. The outcome of the experiments showed that SBCS-YOLOv5s achieved a mean average precision (mAP (0.5:0.95)) of 87.7%, which is 3.5% superior to the original YOLOv5s model. Moreover, SBCS-YOLOv5s has a detection speed of 2.6 ms per image. Compared to other state-of-the-art detection algorithms, SBCS-YOLOv5s performed the best, showing tremendous promise for tomato detection in natural environments.

## 1 Introduction

The tomato is one of the world’s most important crops (Peixoto et al., 2017), but harvesting tomatoes under natural conditions remains labor-intensive. Fruit harvesting has undergone a significant transformation through advances in artificial intelligence within laboratory research. This evolution has paved the way for the emergence of fruit-picking robots anticipated to supplant manual labor. The vision system plays a vital role in a fruit-picking robot. This is because of its fundamental role in accurately identifying fruits, a crucial initial step hinging on the robot’s precision, efficiency, and resilience. Nevertheless, the challenges posed by natural conditions introduce complexities, such as unbalanced lighting, occlusions, overlapping, and other unforeseeable elements (Gongal et al., 2015), all of which affect the detection accuracy of fruit-picking robots. Consequently, enhancing the accuracy, efficiency, and robustness of harvesting robots under these natural conditions is essential.

Many researchers have studied fruit detection to deal with the problems mentioned above. Some digital image processing approaches, such as color features (Goel and Sehgal, 2015; Yang et al., 2020), shape (Jana and Pareskh, 2017), and texture (Rakun et al., 2011), have been proposed to obtain reasonable detection results. Zhao et al. (2016a) developed a technique for detecting immature citrus fruits in natural environments based on cascaded pixel segmentation. A combination of color feature maps and a block-matching method were employed to identify potential fruit pixels. Subsequently, further refinement is adopted utilizing an SVM classifier to eliminate false positives. On the other hand, in the initial stages of segmentation, by relying solely on color features, numerous fruit instances remain undetected due to the resemblance between green fruit and the background. Kurtulmus et al. (2011) introduced a new eigenfruit feature for identifying green citrus. This characteristic was paired with color information and a study of circular Gabor texture. Despite including the texture characteristics alongside color features, their method has encountered challenges distinguishing some fruit from the background and has struggled to detect heavily obscured fruit effectively.

Other methods include K-means clustering (Jiao et al., 2020), Support Vector Machine (SVM) (Azarmdel et al., 2020), and AdaBoost algorithms (Payne et al., 2014). In tomato detection, Liu et al. (2019) developed an approach to identify mature tomatoes within natural environments. A naive Bayesian classifier with an oriented gradient histogram was used to distinguish each tomato. Subsequently, a color analysis step was used to remove false positives. Nevertheless, adapting this method to natural settings posed a challenge owing to the inherent limitations of manually crafted features in terms of their capacity for high-level abstraction. Similarly, Zhao et al. (2016b) used Haar-like feature thresholding and AdaBoost classifier to detect tomatoes. Their study revealed a recognition rate of 96% for tomatoes within their testing dataset. Nevertheless, a long training time was required in their approach.

The aforementioned methods relying on manually designed features have inherent limitations, particularly in scenarios where intricate feature extraction is demanded. The introduction of deep learning successfully addressed these challenges. For example, Rahnemoofar and Sheppard (2017) showcased commendable fruit-counting capabilities through a customized Inception-ResNet architecture (Szegedy et al., 2017). On the other hand, this model focused exclusively on fruit counting and failed to detect them. Santo et al. (2020) introduced a method to detect and track grape clusters within images captured in vineyards. This approach utilized the Mask-RCNN algorithm (He et al., 2017) for the precise detection of individual grape bunches. Furthermore, structure from motion techniques were applied to achieve the 3D alignment of images, enabling the effective mapping of features across various images. Their method achieved an F1-score of 91%. In recent years, the emergence of YOLO models has revolutionized object detection (Redmon et al., 2016; Redmon and Farhadi, 2017; Redmon and Farhadi, 2018; Boschkovskiy et al., 2020; Jocher et al., 2020; Wang et al., 2022). These YOLO models exhibited exceptional improvement in accuracy and speed, surpassing traditional two-stage pipelines (He et al., 2017; Girshick et al., 2014; Ren et al., 2015). They used a single feed-forward network to detect bounding boxes and corresponding classes. Wang et al. (2021) introduced an innovative method anchored in an enhanced YOLOv3-tiny model to identify disease occlusion and overlapping tomato leaves. This model strategically mitigated information loss during network transmission, resulting in a commendable mAP score of 93.1%. Bresilla et al. (2019) used DCNN architectures based on single-stage detectors. Leveraging deep learning techniques eliminates the need to manually code specific features tailored to particular fruit shapes, colors, or other attributes. This method achieved an accuracy of more than 90%. Liu et al. (2020) introduced YOLO-Tomato, a resilient model based on YOLOv3. This model achieved an Average Precision (AP) of 96.40% and a rapid detection speed of 54 ms. Chen et al. (2022) introduced a modified YOLOv4 to detect citrus. Their approach used an attention mechanism and a depth-wise separable convolution module. Moreover, they applied a pruning algorithm to eliminate the impact of irrelevant latent factors in the data. Their average improved from 92.89% to 96.15%, with 0.06s to detect each image.

Expanding the scope, Cao et al. (2023) proposed a technique for persimmon recognition in natural environments. They harnessed an enhanced YOLOv5 model, achieving an average accuracy of 95.53%. Mbouembe et al. (2023) proposed a modified YOLOv4-tiny model for tomato recognition. Their enhancements included a refined backbone design, reducing computational complexity while augmenting feature extraction. A simplified CSP (Cross-Stage Partial Connections) - Spatial Pyramid Pooling was incorporated to improve the receptive field of the backbone. This modification aimed to enhance the ability of the model to capture information from a wider area of the input data. The CARAFE module in the neck network further improved the quality of the feature map. Their method produced an mAP of 82.8%.

**Figure 1:**
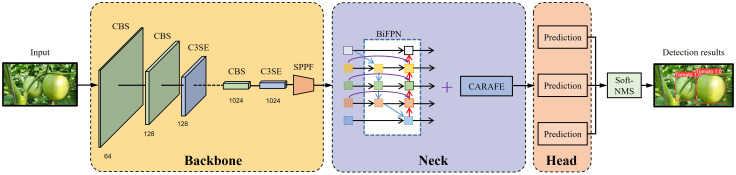
Overview of the SBCS-YOLOv5s.

Despite extensive research in the fruit recognition domain within natural conditions, it is essential to improve the detection accuracy and robustness to fulfill the requirements of fruit detection. This study introduced a precise and resilient tomato detection methodology grounded in the YOLOv5s model to address these persisting challenges. **Figure 1** provides a concise overview of the proposed SBCS-YOLOv5s. The pivotal modifications of this research are outlined as follows:

1. “C3SE Integration”: By amalgamating the SE attention module and the C3 module into a cohesive C3SE module, the conventional C3 module within the YOLOv5s backbone network is upgraded. This integration augments the capacity of the model to provide useful information, bolstering feature extraction.
2. “Bi-directional Feature Pyramid Network Integration”: The original multi-scale PANet feature fusion network is replaced with an efficient weighted Bi-directional Feature Pyramid Network. This alteration enhances feature propagation and reuse, thereby refining overall feature representation.
3. “CARAFE Module Adoption”: Positioned within the network’s neck, the CARAFE module is harnessed to generate an improved feature map enriched with more intricate semantic information.
4. “Soft-NMS Algorithm Implementation”: A noteworthy shift occurs in the detection post-processing stage, where the conventional NMS algorithm yields to the enhanced Soft-NMS algorithm. This transition amplifies the capacity to identify overlapping and occluded fruit.
5. “Performance Evaluation”: Rigorous evaluation using tomato datasets unveils that the proposed SBCS-YOLOv5s model surpasses the original YOLOv5s model and other contemporary update object-detection methods in terms of accuracy.

Focusing on these goals, the study aimed to contribute to advancing tomato harvesting robots by developing an accurate tomato detection model that outperforms existing models in terms of accuracy and efficiency.

## 2 Theoretical background

### 2.1 YOLOv5 network

The YOLOv5s model (Jocher et al., 2020), pioneered by Ultralytics LLC in 2020, is composed of three core components: backbone, neck, and head networks. This study targets the YOLOv5s variant because of its superior performance compared to other iterations within the YOLO series. The backbone network employs a series of convolutional operations and fusion steps to extract the feature maps from input images. Subsequently, the neck network integrates feature maps of diverse dimensions, obtained from the backbone network. This amalgamation yields an upgraded, innovative feature map that effectively preserves contextual information, mitigating information loss. It is important to highlight that this process leverage the FPN (Feature Pyramid Network) structure (Lin et al., 2017) to facilitate the propagation of robust semantic features from higher-level feature maps to their lower-level counterparts. Simultaneously, the PANet (Path Aggregation Network) architecture (Liu et al., 2018) facilitates the transmission of robust localization features from lower-level feature maps to their higher-level counterparts. The head network, the final segment of the model, consists of three layers that generate output feature maps at distinct scales.

The CBS (Convolution, batch normalization, and SiLU activation function) is a conventional convolution layer in the YOLOv5s network. It encompasses a sequence of operations, including convolution, batch normalization (Ioffe and Szegedy, 2015), and the SiLU activation function (Elfwing et al., 2018). YOLOv5 originally employed the BottleneckCSP module instead of the C3 module for feature extraction. The BottleneckCSP module combines the concepts of Bottleneck (He et al., 2016) and CSP (Cross-Stage Partial connections) (Wang et al., 2020a). It involves three successive convolutional kernel operations, with the output of the first being processed through two more convolutional kernels. This sequence culminates in the fusion of unprocessed and convolved features. The primary objective of the BottleneckCSP module is to deepen the model.

The CSP module introduced by Wang et al. (2020a) splits the input into two segments; one undergoes processing via a block (like Bottleneck), while the other proceeds directly through a 1×1 convolutional layer. These two streams are then recombined. The C3 module supplants a 1×1 convolutional layer within the BottleneckCSP module, simplifying the network architecture to enable the extraction of feature maps and minimize the computation complexity. The C3 module comprises two branches, each involving a convolution operation that reduces the feature map channel count by half. The output from these two branches is concatenated using the Bottleneck module, followed by a convolutional layer within the second branch. These processes tightly integrate the output feature maps from both branches, with a final convolutional layer generating the output feature map of the module. Furthermore, SPPF (Spatial Pyramid Pooling Fusion) augments the ability of the backbone to express features. This module employs a sequence of three convolutions with identical kernels, focusing on the amalgamation of features from various resolutions.

### 2.2 Content-aware reassembly of features

The YOLOv5 model uses a nearest neighbor interpolation method for its up-sampling process, utilizing the same kernel for up-sampling across the feature map. Nevertheless, this approach does not leverage the semantic information in the feature map during the up-sampling process, resulting in a significant loss of features. This study integrates the CARAFE module (Wang et al., 2019), a novel technique, to address these limitations. The CARAFE module consists of two main components: a content-aware reassembly module and a kernel prediction module. It anticipates and assembles the recombined kernel, reconstructing the features within predetermined local regions at each point while using the underlying content details. The CARAFE module dynamically adjusts and optimizes the reassembled kernels at distinct points based on the content information, offering superior performance compared to alternative up-sampling methods like interpolation. For every predefined location, the utilization of a reassembly kernel becomes imperative, with the kernel size denoted as *k_up_*. The reassembly procedure is illustrated using (Equation 1):


(1) $$O_{l^{\prime }}=\sum_{n=-r}^{r}\sum_{m=-r}^{r}W_{l_{\left(n,m\right)}^{'}}\cdot I_{\left(i+n,j+m\right)}$$

where *O* and *I* represent the output and input, respectively. $W_{{\text{l}}^{\prime }}$ denotes the location-wise kernel associated with each location $\;l^{'}$ based on the input. $l^{\prime }$ signifies the neighboring location of *l*, and $r=\frac{k_{up}}{2}$ .

**Figure 2:**
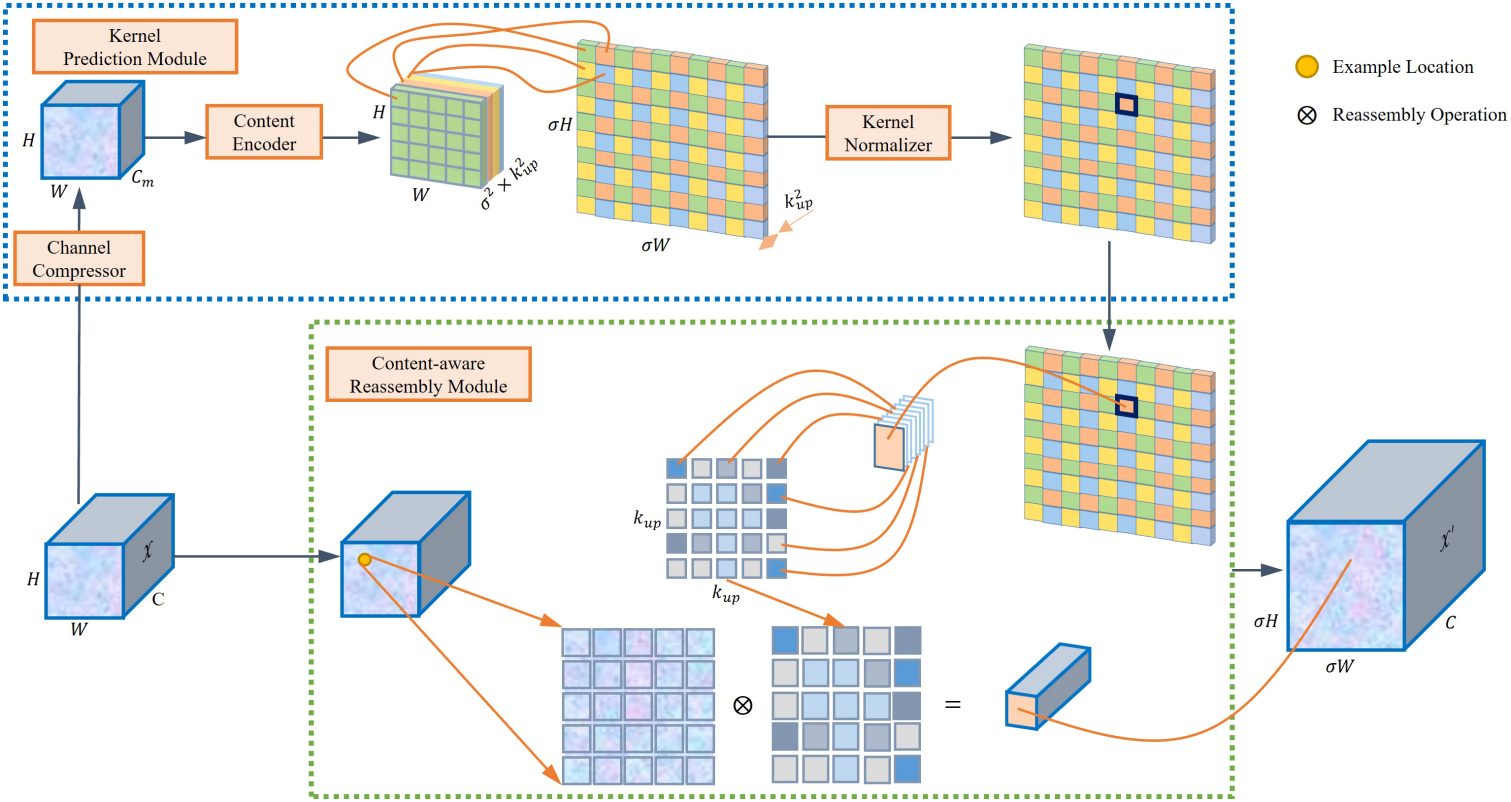
Overall architecture of the CARAFE module.

The CARAFE approach significantly enhances the semantic richness of the reassembled feature maps compared to the nearest neighbor interpolation up-sampling technique. This approach is achieved by strategically emphasizing crucial points within localized regions. In scenarios where tomatoes overlap or are densely packed, CARAFE’s ability to enhance spatial detail helps the model distinguish between closely spaced fruits, potentially reducing the number of merge detections. It also helps the model to improve localization accuracy in tomato detection. In addition, CARAFE encompasses a wider scope of observation, adept content handling, and its lightweight design, culminating in expedited computations. **Figure 2** shows the architectural representation of CARAFE.

## 3 Materials and methods

### 3.1 Image acquisition

Images of tomatoes were taken from December 2017 to November2019 in the greenhouses of a tomato production base, located in Shouguang city, China, with a digital camera (DSC-W170, Sony, Tokyo, Japan) at a resolution of 3648×2056 pixels. The camera was equipped with a 5×Carl Zeiss Vario-Tessar precision zoom lens. The distance between the camera and the target was from 500 mm to 1000 mm. Nine hundred and sixty-six images were captured under natural daylight (sunny and cloudy days) with different conditions such as shading, sunlight, occlusions, and overlaps. The training set had 725 images, while the test set contained 241 images. The scale of tomatoes in the images varies greatly, ranging from 200 to 1500 pixels in diameter.

### 3.2 Image augmentation

**Figure 3:**
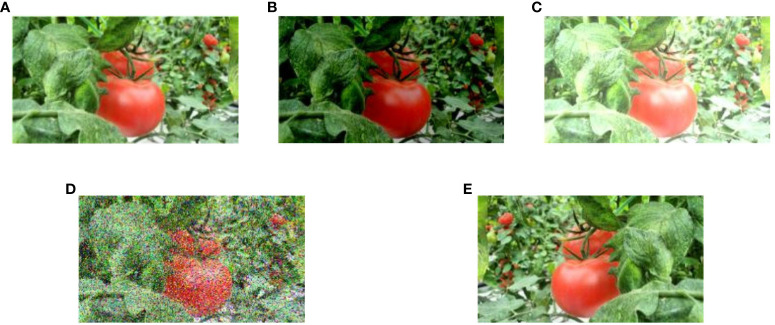
Examples of data enhancement techniques. **(A)** Input image, **(B, C)** varied exposure, **(D)** Noise (salt and pepper), and **(E)** Horizontal Flip.

This study used data augmentation to counteract potential issues, such as over-fitting or non-convergence, that could arise during training. The augmentation of images was accomplished by applying diverse techniques, such as brightness transformation, blur, horizontal flip, noise, and rotation. These methods were employed to enhance the resilience of the model against noise and its ability to remain unaffected by variations in camera positioning. In particular, introducing a Random Gaussian blur makes the model more resistant to camera blur, with a threshold of 25 pixels for maximum blur. In addition, the incorporation of horizontal and vertical flips played a role in fortifying the capacity of the model to perform consistently regardless of the orientation of the subject. A visual representation of data enhancement techniques can be observed in **Figure 3**.

### 3.3 The SBCS-YOLOv5s architecture

**Figure 4:**
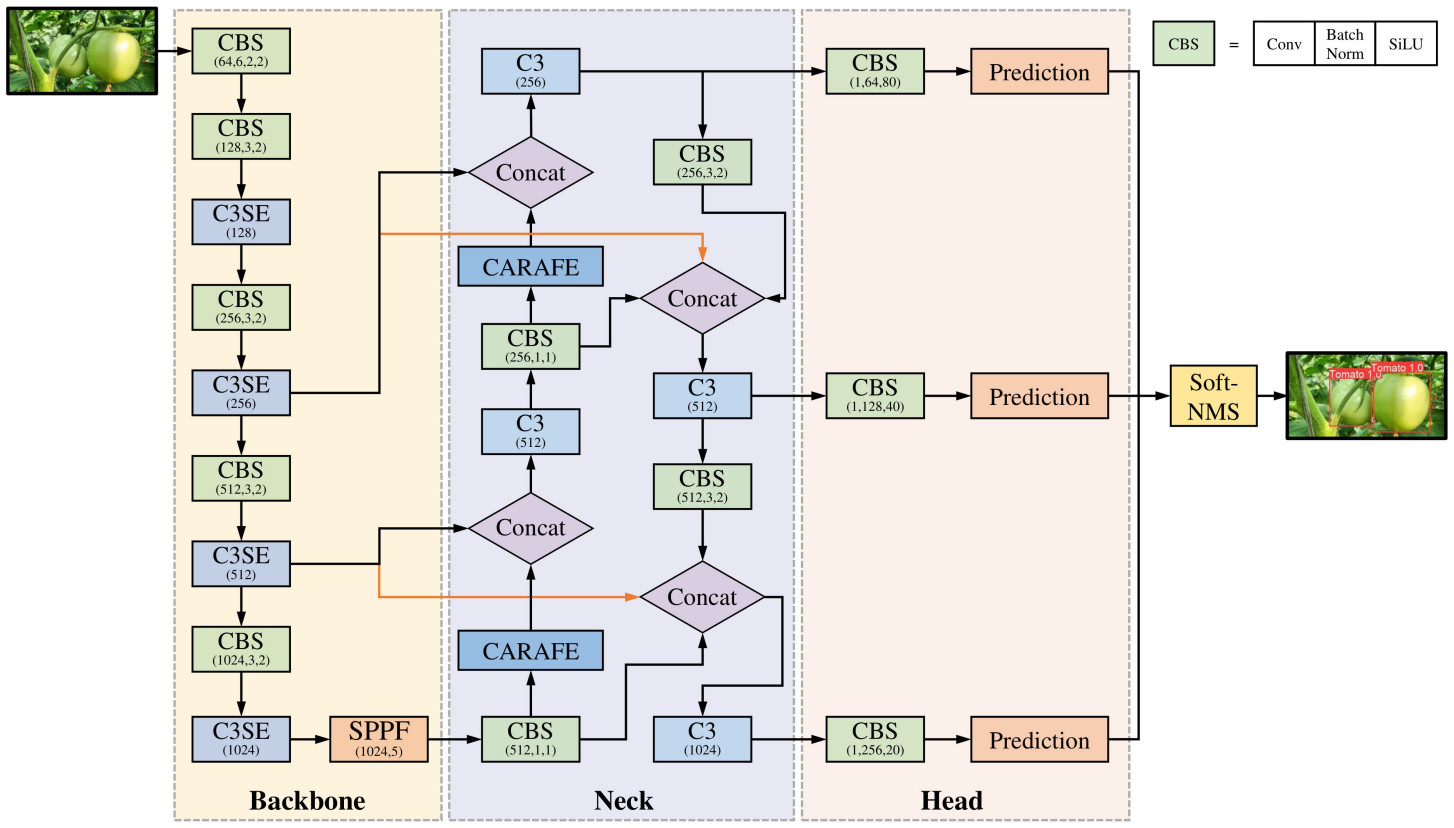
The architecture of SBCS-YOLOv5s.

The YOLOv5 model represents a single-stage object detection algorithm that introduces substantial enhancements over other YOLO models. On the other hand, the challenge of achieving high accuracy and fast speed persists in the tomato detection case, primarily because of the intricacies of the natural environment, such as occlusions and overlapping. This study proposes an SBCS-YOLOv5s model to address this issue, with the incorporation of SE, BiFPN, CARAFE, Soft-NMS into the YOLOv5s. The first module of this approach is used for feature extraction, merging the SE module (Hu et al., 2018) and the C3 module of the YOLOv5s model. This fusion enhances the network focus on useful information, refines the feature extraction process, and improves the model’s robustness to variations in environmental conditions. The neck network integrates BiFPN (Tan et al., 2020) and CARAFE modules (Wang et al., 2019) into YOLOv5s, enriching features with more profound semantic information. The conventional NMS algorithm (Hosang et al., 2017) used in YOLOv5s was substituted with the Soft-NMS algorithm (Bodla et al., 2017) to make the network more efficient in detecting occluded and overlapped fruits. Additional intricacies of this approach are elaborated upon in subsequent sections. **Figure 4** presents the architecture of SBCS-YOLOv5s.

#### 3.3.1 The modified backbone network

**Figure 5:**
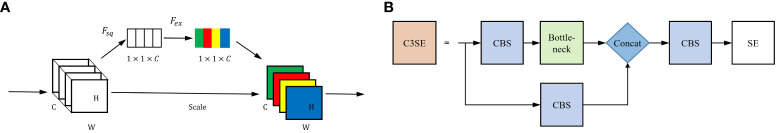
Original SE module and improved C3SE module architecture. **(A)** SE module architecture, **(B)** C3SE module architecture.

The SE attention module (Hu et al., 2018) in **Figure 5A** is fused with the C3 module structure into an improved C3SE module. The SE module solves the issue of feature maps containing informative and less relevant channels. The re-calibration process empowers the network to prioritize informative channels while suppressing the less useful ones. In addition, the SE module’s ability to adaptively re-calibrate features helps to improve the robustness of the model to variations in illumination and environmental conditions. It also helps to reduce over-fitting, which is essential for tomato detection to accurately identify the boundaries of individual tomatoes in an image. **Figure 5B** presents the structure of the C3SE module. The weight of each channel is allocated using the interdependence of the feature channels to facilitate the neural network to focus on significant feature information and to minimize the impact of feature redundancy. The SE attention module comprises three key operations: squeeze, excitation, and scale.

The squeeze operation, also called compression, involves applying a global average pooling operation to each channel of the feature map. This compresses the spatial dimensions of the feature map, converting its size into multiple features while maintaining the overall channel dimension. For example, if the input feature map holds a size of *H*×*W*×*C*, and $V=\left[v_{1},v_{2},\;\ldots ,\;v_{c}\right]$ is an example input set, the transformation of the squeeze operation can be expressed using (Equation 2).


(2) $$F_{sq}\left(V_{c}\right)=\frac{1}{H\times W}\sum_{i=1}^{H}\sum_{j=1}^{W}V_{c}\left(i,j\right)$$

where c∈C , and *C* denotes the number of feature channels, while *W* signifies the feature map width; *H* corresponds to the height of the feature map; $F_{sq}$ denotes the specific squeeze operation being discussed.

The excitation operation consists of two primary components: a fully connected layer and a sigmoid activation function. The fully connected layer incorporates comprehensive information from all input features. Subsequently, the sigmoid function transforms the input into a range confined within [0,1]. This process is visually represented by (Equation 3).


(3) $$F_{ex}\left(F_{sq},B\right)=\sigma (B_{1}\cdot \delta \left(B_{2}\cdot F_{sq}\right)$$

where σ symbolizes the sigmoid activation function, δ signifies the ReLU activation function, and *F_ex_* denotes the excitation operation. *B*_1_ and *B*_2_ denote the weights of the fully connected layer, respectively.

Finally, the scale operation combines or multiplies the input channel weight with the weight derived from the channel feature of the two preceding operations. (Equation 4) shows the rescaling operation:


(4) $$F_{scale}\left(V_{c},S_{c}\right)=S_{c}V_{c}$$

where $F_{scale}\left(V_{c},S_{c}\right)$ refers to channel-wise multiplication that takes place between *S_c_* and *V_c_*.

#### 3.3.2 The modified neck network

**Figure 6:**
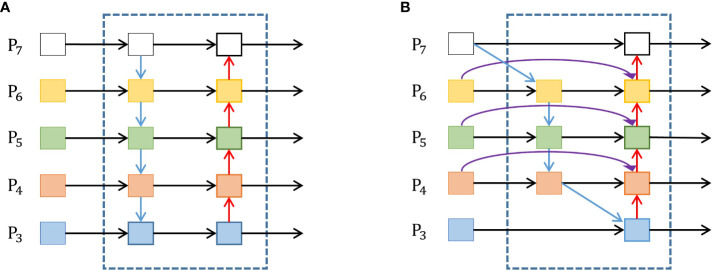
Architectures of PANet and BiFPN. **(A)** PANet architecture, **(B)** BiFPN architecture.

The FPN+PANet network was replaced in the YOLOV5s neck with the weighted BiFPN in this study. The rationale stems from large-scale objects possessing many pixels, whereas small objects have few. The features of large objects can be easily maintained in the convolution process, while the features of the smaller ones can be easily ignored. The YOLOv3 model introduced the FPN network structure (Lin et al., 2017), emphasizing the down-sampling process of semantic information extraction. Based on this, the YOLOv5 incorporates PANet (Liu et al., 2018) to aggregate image features by incorporating secondary bottom-up fusion, as shown in **Figure 6A**. This approach integrates accurate low-level localization signals to enrich the entire feature hierarchy and facilitate the flow of information. On the other hand, PANet is characterized by simple two-way fusion, and their contributions to the output features often remain unequal because of the varying input resolutions. Furthermore, feature fusion of PANet involves a direct addition of distinct input features, leading to unbalanced output features and complicating computational processes.

The BiFPN, introduced by Tan et al. (2020), is an object detection model module. Its main strength lies in effectively fusing information within a deep learning network, ensuring efficiency and accuracy. The problem of correctly combining multi-scale features from multiple layers of a convolutional neural network are solved to improve the detection accuracy of objects at various scales. The bottom-up and top-down paths are used to construct a feature pyramid that captures fine-grained features. The BiFPN combines the feature maps from the bottom-up and top-down paths. Furthermore, to avoid all feature maps contributing equally, the BiFPN provides learnable weights for each input feature map, allowing the network to assign varied priorities to different scales and resolutions. The notable enhancement brought by BiFPN is the introduction of a bi-directional connection between neighboring levels of the network. This augmentation substantially improves the flow of information and gradient propagation during the training process. It also improves to tomato localization, helping the model to capture details at different scales and make more accurate predictions of tomato locations. In addition, BiFPN is designed to be computationally efficient, making it well suited for real-time detection. **Figure 6B** shows the BiFPN architecture. (Equation 5) shows the fast normalized fusion between the feature maps from the bottom-up and top-down paths.


(5) $$\{\begin{array}{c} P_{6}^{td}=Conv(\frac{w_{1}\cdot P_{6}^{in}+w_{2}\cdot Resize\left(P_{7}^{in}\right)}{w_{1}+w_{2}+\varepsilon }\\ P_{6}^{out}=Conv(\frac{w_{1}^{\prime }\cdot P_{6}^{in}+w_{2}^{\prime }\cdot P_{6}^{td}+w_{3}^{\prime }\cdot Resize\left(P_{5}^{out}\right)}{w_{1}^{\prime }+w_{2}^{\prime }+w_{3}^{\prime }+\varepsilon } \end{array}$$

where the intermediate feature situated at Level 6 along the top-down pathway is $P_{6}^{td}$ , while the resulting feature at Level 6 stemming from the bottom-up pathway is $P_{6}^{out}$ , *Conv* and *Resize* correspond to convolution and sampling operations, respectively. *w* and ϵ represent the weight and a small pre-set value to avoid numerical instability, respectively. Usually, this value was set to 0.0001.

BiFPN improves the detection accuracy compared to the PANet used in the YOLOv5s model. Nevertheless, the BiFPN employs a nearest neighbor interpolation method for the up-sampling of feature maps. Using this approach could lead to a small receptive field and make the network focus only on sub-pixel spaces, resulting in the loss of rich semantic information. In this study, the CARAFE module was introduced to the BiFPN to tackle this problem. This integration improved feature maps with rich information and high resolutions. Section 2.2 describes the CARAFE module in detail.

#### 3.3.3 Soft-NMS (non-maximum suppression) algorithm

The soft-NMS algorithm (Bodla et al., 2017) is a modified version of the conventional NMS algorithm (Hosang et al., 2017) used by the YOLOv5 framework. The fundamental principle behind the NMS algorithm involves selecting the bounding box with the highest confidence score. It suppresses the other bounding boxes with significant overlap with the selected box, leading to the missed detection of overlapping fruits. Moreover, the NMS algorithm does not perform optimally when dealing with different scales. Equation 6 shows the NMS algorithm:


(6) $${\hat{S}}_{i}=\{\begin{array}{c} {\hat{S}}_{i},\;IoU\;\left(M,{\hat{b}}_{i}\right)<N_{t}\\ 0,\;IoU\;\left(M,{\hat{b}}_{i}\right)\;\geq \;N_{t} \end{array}$$

where ${\hat{b}}_{i}$ and ${\hat{S}}_{i}$ denote the i th predictor and its score, respectively. *N_t_* is the pre-set threshold; *M* denotes the candidate box having the highest score; $IoU\;\left(M,{\hat{b}}_{i}\right)$ is the overlap region between *M* and ${\hat{b}}_{i}$ .

The objective of the Soft-NMS algorithm is to solve the limitations of the traditional NMS algorithm approach. It is also designed to be more tolerant to overlapping objects. This is achieved using a softening function that progressively decreases the scores of bounding boxes overlapping with the one possessing the highest score. The primary goal is to reduce the severe suppression of surrounding boxes that might be slightly less confident but still contain useful information. This modification seeks to enhance the detection accuracy and improve the handling of cases involving overlapping fruits within the final detection results. And it helps maintain a consistent ranking of bounding box scores, even when there is overlap. The Soft-NMS algorithm is outlined in (Equation 7):


(7) $${\hat{S}}_{i}=\{\begin{array}{c} {\hat{S}}_{i},\;\;\;\;\;\;\;IoU\;\left(M,{\hat{b}}_{i}\right)<N_{t}\\ {\hat{S}}_{i}e^{-\frac{IoU{\left(M,{\hat{b}}_{i}\right)}^{2}}{\sigma }},\;IoU\;\left(M,{\hat{b}}_{i}\right)\;\geq \;N_{t} \end{array}$$

where  σ represents the hyperparameter of the penalty function. When the $\;IoU\;\left(M,{\hat{b}}_{i}\right)$ exceeds the pre-defined threshold, the prediction frame confidence score is reduced systematically instead of being set to zero. As a result, the detection accuracy of overlapping and occluded fruits can be improved.

#### 3.3.4 Loss function

The loss function used in this study is expressed as (Equation 8), which encompasses the regression error of bounding coordinates, the confidence error of the bounding box, and the classification error of object category.


(8) $$L=Loss_{reg}+Loss_{conf}+Loss_{cls}$$

In this study, the bounding box regression loss incorporatesthe use of CIoU (Complete IoU) as in (Equation 8.a). It could accurately represent the gap between the prediction and annotation frames, enhancing the network model during training. It also considers crucial factors, such as the overlapping area (expressed in Equation 8.b), central point distance, and aspect ratio (expressed in Equation 8.c) between b and $b_{ɡt}$ .


(8.a) $$CIoU=1-IoU+\frac{d^{2}\;\left(\hat{b},b_{ɡt}\right)}{c^{2}}+\alpha v$$

with


(8.b) $$IoU=\;\frac{\hat{b}\cap^{\;}b_{ɡt}}{\hat{b}\cup^{\;}b_{ɡt}}$$

and


(8.c) $$v=\frac{4}{\pi^{2}}{\left(tan^{-1}\frac{w_{ɡt}}{h_{ɡt}}-tan^{-1}\frac{w}{h}\right)}^{2},\;\alpha =\frac{v}{\left(1-IoU\right)+v}$$

where *b* and *b_ɡt_* represent the predicted and ground truth bounding boxes, respectively. *d* signifies the distance between the predicted center point and the true center point; *c* is the diagonal length of the enclosing box covering *b* and *b_ɡt_*; *α* and *v* are the positive trade-off and aspect ratio parameters, respectively.

Object classification loss is expressed as (Equation 8.e), wherein the process is initiated by calculating the confidence *C* of the cell grid as in Equation 8.d):


(8.d) $$C=P\left(object\right)\times IoU\left(b,b_{ɡt}\right)$$

then,


(8.e) $$\begin{array}{l} Loss_{conf}=\sum_{i=1}^{s\times s}\sum_{j=1}^{NB}\lambda_{i,j}[C_{i}\cdot log({\tilde{C}}_{i})log\left(1-C_{i}\right)]\\ \;-\sum_{i=i}^{s\times s}\sum_{j=1}^{NB}\left(1-\lambda_{i,j}\right)[C_{i}\cdot log{\tilde{C}}_{i}+\left(1-C_{i}\right)log\left(1-{\tilde{C}}_{i}\right) \end{array}$$

with λi,j expresses in (Equation 8.f):


(8.f) $$\lambda_{i,j}=\{\begin{array}{c} 1,\;if\;part\;of\;j-th\;bounding\;box\;is\;in\;the\;i-th\;grid\;cell\\ 0,\;otherwise \end{array}$$

where s×s denotes the size of the grid cell; *NB* stands for the number of bounding boxes; ${\tilde{C}}_{i}$ represents the confidence obtained from the prediction box; *C_i_* signifies the confidence threshold.

### 3.4 Experimental setup

The experiments of this research were conducted using an Intel i5 64-bit quad-core CPUs operating at a frequency of 3.30 GHz (Santa Clara, CA, USA). The system had 16 GB of RAM and an NVIDIA GeForce GTX 1070Ti GPU with 8 GB memory. The chosen model framework was PyTorch, with CUDA 11.1 and Python 3.8.10 for implementation. **Table 1** lists some hyper parameters used in the experiments.

**Table 1:** Configuration of certain hyper-parameters.

Parameters	Value
Number of epochs	400
Learning rate	0.001
Optimizer weight decay	94.75
STD momentum	96.3
Warm-up initial momentum	0.8
Batch size	8
Box loss gain	0.05
Cls (classification loss gain)	0.5
Cls_pw (cls BCE loss positive weight)	1.0
Obj (object loss gain)	1.0
Anchor_t (anchor multiple threshold)	4.0

The criteria used for assessing the performance of fruit detection encompassed precision, recall, mean average precision (mAP), and *F*_1_ score (Padilla et al., 2020). The metrics are defined in (Equations 9–12):


(9) $$R=\frac{True\;Positive}{True\;Positive+False\;Negatiive}$$

(10) $$P=\frac{True\;Positive}{True\;Positive+False\;Positive}$$

where *R* and *P* are the recall and precision, respectively. Using mAP is a valuable approach to assess the model performance across different confidence levels.


(11) $$mAP=\frac{1}{N_{cls}}\sum_{a=1\;}^{N_{cls}}AP_{a}$$

with *AP* expresses in (Equation 11.a):


(11.a) $$AP=\sum_{q}^{Q}\left(r_{q+1}-r_{q}\right)\underset{\tilde{r}\geq r_{q+1}}{\max}p\left(\tilde{r}\right)$$

where $p\left(\tilde{r}\right)$ represents the calculated precision at a given recall value ( $\tilde{r}$ ), while $N_{cls}$ is the total number of classes.


(12) $$F_{1}=\frac{2\times R\times P}{R+P}$$

## 4 Results and discussions

### 4.1 Ablation study

The first step in this study was to determine which attention mechanism (CBAM (Woo et al., 2018), ECA (Wang et al., 2020b), CA (Hou et al., 2021), SE (Hu et al., 2018)) works better on the tomato datasets after fusing the original C3 module network. From **Table 2**, we can see that integrating the SE attention module with the C3 module led to a notable outcome. The mean average precision with an IoU of 0.5 to 0.95 reached 85.1%, which is the best result.

**Table 2:** Ablation analysis of different attention mechanisms.

	C3	CBAM	ECA	CA	SE	mAP (0.5:0.95) (%)
Modifications	**✓**					84.2
	**✓**	**✓**				83.7
	**✓**		**✓**			84.9
	**✓**			**✓**		84.4
	**✓**				**✓**	85.1

Since the SE attention module relies on modeling channel-wise relationships and adaptive re-calibration of feature maps to capture important information, it helps to improve feature extraction of the model. The fusion of the SE attention module with the C3 module was implemented within the backbone network. Furthermore, the integration of BiFPN, CARAFE, and Soft-NMS was used in the neck to improve the detection capabilities of YOLOv5s. An ablation study was carried out to evaluate the effectiveness of each component.

Integrating the SE attention module with the C3 module resulted in a 0.9% increase in the mean average precision with an IoU of 0.5 to 0.95, as shown in **Table 3**. This enhancement underscores the efficacy of the SE attention module to channel the model towards useful information. Subsequently, a further increase of 0.6% in mAP was achieved by replacing PANet with BiFPN. This is because the BiFPN assists the model in determining useful weights for comprehensive fusion of high-level and low-level features, thereby improving detection performance. Discernible performance improvements became evident after incorporating the CARAFE module as an up-sampling operator within PANet and BiFPN. This is due to the fact that CARAFE enhances spatial details and improves feature map resolution better than the original up-sampling method. On the other hand, the most remarkable results emerged when the Soft-NMS algorithm was applied to the BiFPN+CARAFE configuration, showcasing 3.5% advancement over the original YOLOv5s model. This proves the advantage of the continuous weighting scheme of Soft-NMS. This sequence of observations indicates a substantial enhancement in detection performance through different modifications.

**Table 3:** Ablation analysis of different components.

	C3SE	PANet	BiFPN	CARAFE	Soft_NMS	mAP (0.5:0.95) (%)
Modifications		**✓**				84.2
	**✓**	**✓**				85.1
	**✓**		**✓**			85.7
	**✓**	**✓**		**✓**		86.7
	**✓**		**✓**	**✓**		87.2
	**✓**		**✓**	**✓**	**✓**	87.7

### 4.2 Feature map visualization

**Figure 7:**
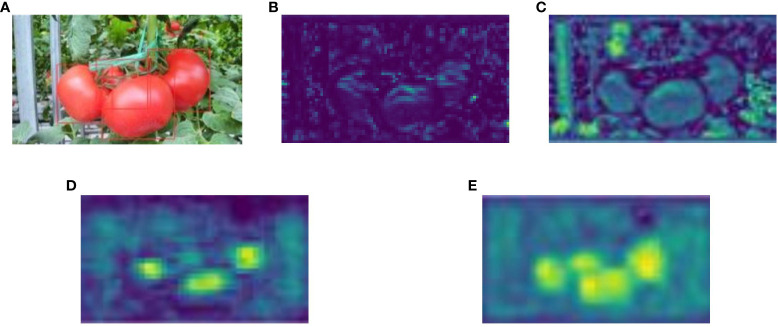
**(A)** Annotated input image, **(B)** the feature of the C3 module of YOLOv5s, **(C)** feature of the C3SE module of SBCS-YOLOv5s, **(D)** feature of the original neck of YOLOv5s, and **(E)** feature of neck of SBCS-YOLOv5s.

Visualizations were performed to compare the improved model variants with the original YOLOv5s. **Figure 7A** presents an input image with tomatoes annotated for improved visibility. **Figures 7B, C** show the difference between the C3 and C3SE modules, respectively. In particular, **Figure 7C** highlights finer details that are more discernible. This observation underscores the role of the SE module in steering the backbone network towards useful information. **Figures 7D**, **E** represent the original neck of YOLOv5s and the modified neck used in SBCS-YOLov5s, respectively. **Figure 7E** shows an improved feature map with heightened resolution after incorporating the BiFPN and CARAFE modules. These enhancements facilitate efficient context information aggregation and seamless fusion within the network.

Every modification produced superior features with high resolution compared to those in the original model (**Figure 7**). This visual evidence substantiates that SBCS-YOLOv5s excels in accuracy, resilience, and efficiency when compared to the original model.

### 4.3 Comparison of the SBCS-YOLOv5s with other detection algorithms

The performance of SBCS-YOLOv5s was compared with several other object detection models. These models included Faster-RCNN (Ren et al., 2015), Dynamic-RCNN (Zhang et al., 2020), YOLOv3 (Redmon and Farhadi, 2018), YOLOv3-tiny (Redmon and Farhadi, 2018), YOLOv4 (Boschkovskiy et al., 2020), YOLOv4-tiny (Boschkovskiy et al., 2020), YOLOv7-tiny (Wang et al., 2022), and YOLOv5s (Jocher et al., 2020).

The mean average precision with IoU of 0.5 to 0.95 was 3.8%, 9.7%, 5.8%, 16.4%, 4.6%, 9.3%, 4.5%, and 3.5% higher than those of Faster-RCNN, ynamic RCNN, YOLOv3, YOLOv3-tiny, YOLOv4, YOLOv4-tiny, YOLOv7-tiny, and YOLOv5s, respectively (**Table 4**). Furthermore, the detection time achieved 2.6 ms per image, fulfilling the real-time detection criteria. Moreover, the precision of the proposed model improved by 0.3%, 1.5%, 2.5%, 1.7%, 1.4%, 1.5%, 0.6%, and 1.4% compared to the Faster RCNN, Dynamic RCNN, YOLOv3, YOLOv3-tiny, YOLOv4, YOLOv4-tiny, YOLOv7-tiny, and YOLOv5s, respectively. The F1 score and mAP with an IoU of 0.5 increased by 1.64% and 0.5%, respectively, compared to the original YOLOv5s model. Hence, the performance of SCBS-YOLOv5s was improved compared to other object detection networks. Importantly, the experimental results revealed the efficient real-time detection capability of SCBS-YOLOv5s in accurately identifying tomatoes within their natural environmental context.

**Table 4:** Comparison of the different models.

Model	Precision (%)	Recall (%)	F1 (%)	mAP (0.5) (%)	mAP (0.5:0.95) (%)	Time (ms)
Faster-RCNN (VGG-16)	96.5	94.8	95.6	97.8	83.9	3.9
Dynamic RCNN	95.3	93.2	94.2	96.6	78.0	2.4
YOLOv3	94.3	92.4	93.4	97.1	81.8	4.8
YOLOv3-tiny	95.1	91.9	93.4	97.4	71.3	3.8
YOLOv4	95.4	95.3	95.3	97.5	83.1	4.3
YOLOv4-tiny	95.3	94.0	94.6	98.0	78.4	3.5
YOLOv7-tiny	96.2	94.2	95.1	98.2	83.2	4.3
YOLOv5s^*^	95.4	94.5	95.4	98.2	84.2	4.1
SBCS-YOLOv5s	96.8	97.3	97.04	98.7	87.7	2.6

^*^YOLOv5s v6. 1 version is used in this study.

**Figure 8:**
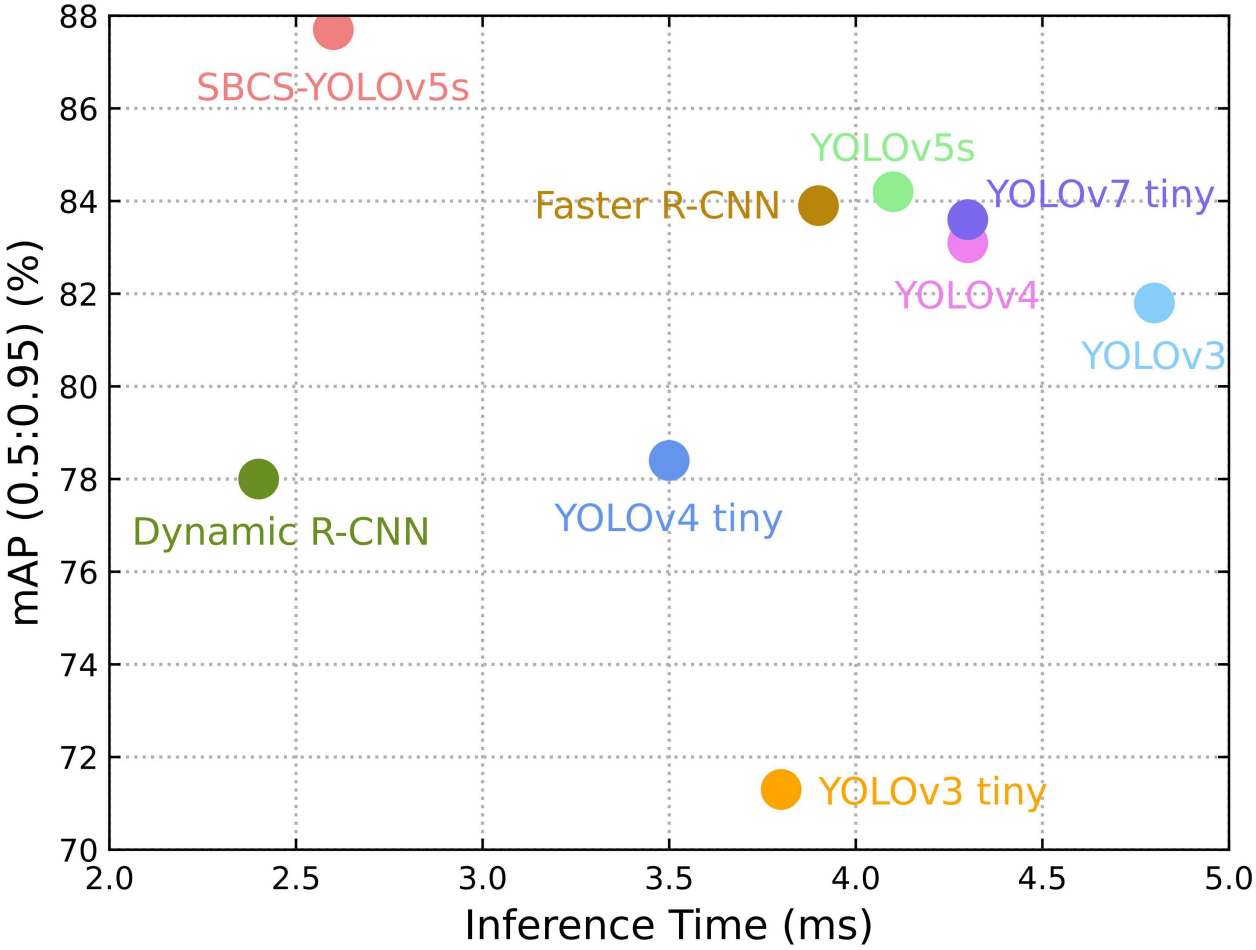
Detection performance of different models (accuracy vs. inference time).

The detection performance of the improved YOLOv5s surpassed that of alternative models while demonstrating greater efficiency (**Figure 8**). The mean average precision with an IoU of 0.5 to 0.95 exhibited a notable 3.5% improvement compared to the original YOLOv5s model. Furthermore, the processing time for detecting each image was decreased by 1.5ms. These results collectively signify the improved model prowess in achieving improved accuracy, compactness, and efficiency when tasked with fruit detection in a natural environment.

### 4.4 Performance of the improved model under different conditions

In a natural environment, tomatoes are exposed to different lighting conditions because of the uneven illumination. Moreover, they can become obscured by leaves or branches and might overlap. The performance of the improved model was assessed across diverse scenarios. **Table 5** shows how the tomatoes were classified into sunshine and shade cases regarding illumination. Within the test dataset, there were 425 tomatoes under shaded conditions and 487 tomatoes under sunlight conditions. In terms of obscured and overlapped severity, the tomatoes were classified as mild and significant occlusion, as delineated in **Table 5**. The latter pertains to situations where tomatoes are obstructed by leaves, branches, or other tomatoes by over 50%.

**Table 5:** Performance of the improved model under different conditions.

Conditions	Tomato Count	Correctly Identified		Falsely Identified		Missed	
		Amount	Rate (%)	Amount	Rate (%)	Amount	Rate (%)
Sunlight	487	473	97.2	15	3.1	14	2.8
Shading	425	414	97.4	14	3.3	11	2.6
Mild occlusion	609	595	97.7	17	2.8	14	2.3
Severe occlusion	303	292	96.4	12	3.9	11	3.6

**Figure 9:**
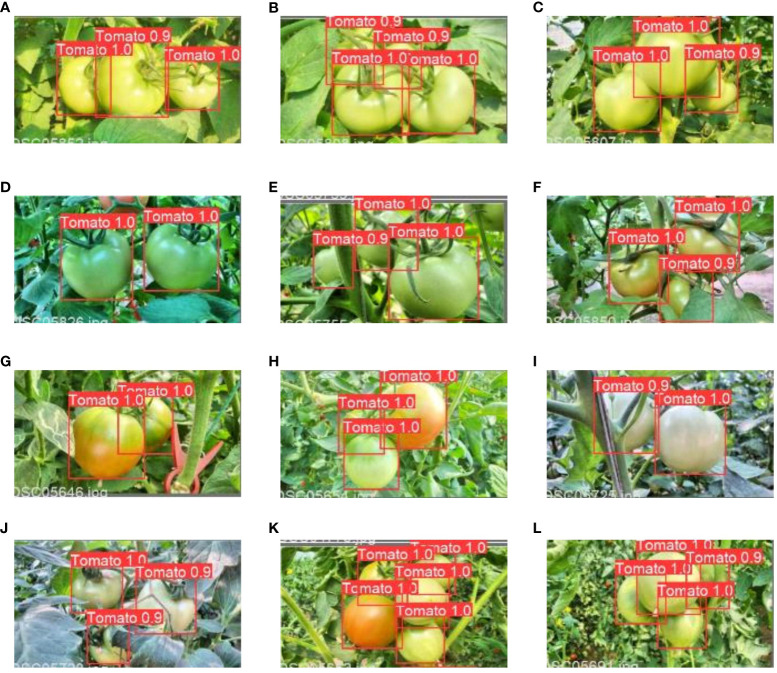
Some examples of tomato detection results under different conditions. **(A–C)** sunlight cases, and **(D–F)** shade cases, **(G–I)** slight occlusions, and **(J–L)** severe occlusions.

The correct detection rate for tomatoes under sunlight conditions was 97.2%, while the rate was 97.4% when tomatoes were in shaded conditions (**Table 5**). False identification was 3.1% for sunlight and 3.3% for shade, respectively. Approximately 97.7% of the tomatoes were detected accurately when they exhibited mild occlusion, with a correctness rate of 96.4% in the case of severe occlusion (**Table 5**). The rates of missed identification were 2.3% and 3.6% for mild and severe occlusions, respectively. **Figure 9** presents some examples of detection outcome instances under various conditions. The results revealed the robustness of the improved model in effectively managing variations in illumination and situations involving overlapping fruits.

### 4.5 Qualitative analysis between SBCS-YOLOv5s and the original YOLOv5s model

**Figure 10:**
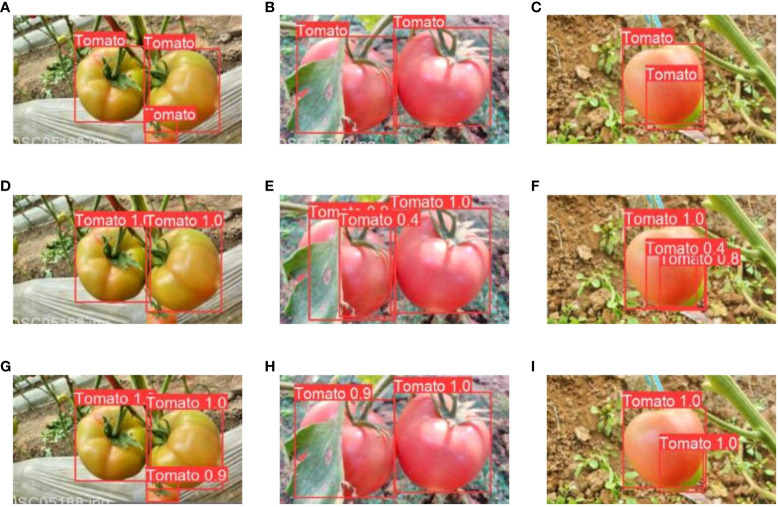
Some detection results from the two models. **(A–C)** ground Truth, **(D–F)** prediction images from the YOLOv5s model, and **(G–I)** prediction images from SBCS-YOLOv5s.

**Figure 10** shows some prediction results from the SBCS-YOLOv5s and the original YOLOv5s model.

As shown in **Figure 10**, the detection performance of SBCS-YOLOv5s was superior to the original YOLOv5s model. In particular, **Figure 10G** visually demonstrates the improved model focus on more useful information and retain the features for small tomatoes. Moreover, the original YOLOv5s model returned some false negatives and false positives, as shown in **Figures 10E**, **F**.

## 5 Conclusions and future work

This paper introduced an accurate and efficient tomato detection solution named SBCS-YOLOv5s, which builds upon the YOLOv5s framework. The accuracy and efficiency of the model were improved by substituting the original C3 module within YOLOv5s with a C3SE module, combining the SE attention module with the C3 module. This change amplified the feature extraction capabilities. Furthermore, the PANet in the neck of the original model was replaced with a weighted Bi-directional Feature Pyramid Network (BiFPN), enhancing the adaptability of the detector to objects of varying scales by fusing high-level and bottom-level features at high resolution. Furthermore, the traditional up-sampling operator within the BiFPN structure was substituted with the CARAFE module to yield more refined semantic information. Finally, the conventional NMS algorithm was replaced with the Soft-NMS algorithm to improve the detection accuracy of overlapped and occluded fruits.

A thorough experimentation was carried out to validate the performance of SBCS-YOLOv5s. An ablation study was instrumental in substantiating the efficacy of each modification. The findings of the experiment showed that the mAP with an IoU of 0.5 to 0.95 had 3.8%, 9.7%, 5.8%, 16.4%, 4.6%, 9.3%, 4.5%, and 3.5% improvements compared to other object detection algorithms, reaching 2.6ms per image in terms of detection time.

Furthermore, the experiments underscored the robustness of SBCS-YOLOv5s because it effectively detected tomatoes across diverse scenarios involving varying lighting and occlusion conditions.

Despite the excellent performance of the improved model, there is room for enhancing the detection performance. In the future study, the explicit incorporation of contextual information will be explored to refine the detection accuracy. Moreover, we will consider incorporating information about tomato maturity to enable differentiation among tomatoes at distinct growth stages based on SBCS-YOLOv5s presented in this study.

## Data availability statement

The original contributions presented in the study are included in the article/supplementary aterial. Further inquiries can be directed to the corresponding authors.

## Author contributions

PT: Conceptualization, Formal analysis, Investigation, Methodology, Software, Visualization, Writing – original draft, Writing – review & editing. GL: Data curation, Formal analysis, Funding acquisition, Software, Validation, Writing – review & editing. SP: Funding acquisition, Investigation, Project administration, Supervision, Validation, Writing – review & editing. JK: Methodology, Supervision, Validation, Writing – review & editing.

## References

[B1] Azarmdel H. Jahanbakhshi A. Mohtasebi S. S. Munoz A. R. (2020). Evaluation of image processing technique as an expert system in mulberry fruit grading based on ripeness level using artificial neural networks (ANNs) and support vector machine (SVM). Posthavest Biol. Technol. 166, 111201. doi: 10.1016/j.postharvbio.2020.111201

[B2] Bodla N. Singh B. Chellappa R. Davis L. S. (2017). “Soft-NMS–improving object detection with one line of code,” in Proceedings of the IEEE International Conference on Computer Vision (ICCV). (Venice, Italy: IEEE),5561–5569. doi: 10.48550/arXiv.1704.04503

[B3] Boschkovskiy A. Wang C. Y. Liao H. Y. M. (2020). YOLOv4: optimal speed and accuracy of object detection. arXiv preprint arXiv:2004.10934. doi: 10.48550/arXiv.2004.10934

[B4] Bresilla K. Perulli G. D. Boini A. Morandi B. Corelli Grappadelli L. Manfrini L. (2019). Single-shot convolution neural networks for real-time fruit detection within the tree. Front. Plant Sci. 10. doi: 10.3389/fpls.2019.00611 PMC6537632 31178875

[B5] Cao Z. Mei F. Zhang D. Liu B. Wang Y. Hou W. (2023). Recognition and detection of persimmon in a natural environment based on an improved YOLOv5 model. Electronics 12, 785. doi: 10.33990/electronics12040785

[B6] Chen W. Lu S. Liu B. Chen M. Li G. Qian T. (2022). CitrusYOLO: a lagorithm for citrus detection under orchard environment based on YOLOv4. Multimed. Tools Appl. 81, 31363–31389. doi: 10.1007/s11042-022-12687-5

[B7] Elfwing S. Uchibe E. Doya K. (2018). Sigmoid-weighted linear units for neural network function approximation in reinforcement learning. Neural Netw. 107, 3–11. doi: 10.1016/j.neunet.201712.012 29395652

[B8] Girshick R. Donahue J. Darrell T. Malik J. (2014). “Rich feature hierarchies for accurate object detection and semantic segmentation,” in Proceedings of the IEEE Conference on Computer Vision and Pattern Recognition (CVPR). (Columbus, OH, USA: IEEE),580–587. doi: 10.48550/arXiv.1311.2524

[B9] Goel N. Sehgal P. (2015). Fuzzy classification of pre-harvest tomatoes for ripeness estimation-an approach based on automatic rule learning using decision tree. Appl. Soft Comput. 36, 45–56. doi: 10.1016/j.asoc.2015.07.009

[B10] Gongal A. Amatya S. Karkee M. Zhang Q. Lewis K. (2015). Sensors and systems for fruit detection and localization: a review. Compt Electr. Agric. 116, 8–19. doi: 10.1016/j.compag.2015.05.021

[B11] He K. Gkioxari G. Dollár P. Girshick R. (2017). “Mask R-CNN,” in Proceedings of the IEEE International Conference on Computer Vision. 2961–2969. doi: 10.48550/arXiv.1703.06870

[B12] He K. Zhang X. Ren S. Sun J. (2016). “Deep residual learning for image recognition,” in Proceedings of the IEEE Conference on Computer Vision and Pattern Recognition (CVPR). (Las Vegas, NV, USA: IEEE), 770–778. doi: 10.1109/CVPR.2016.90

[B13] Hosang J. Benenson R. Schiele B. (2017). “Learning non-maximum suppression,” in Proceedings of the IEEE Conference on Computer Vision and Pattern Recognition (CVPR). (Honolulu, HI, USA: IEEE), pp. 4507–4515. doi: 10.1109/CVPR.2017.685

[B14] Hou Q. Zhou D. Feng J. (2021). “Coordinate attention for efficient mobile network design,” in Proceedings of the IEEE/CVF Conference on Computer Vision and Pattern Recognition (CVPR). (Nashville, TN, USA: IEEE), 13713–13722. doi: 10.48550/arXiv.2103.02907

[B15] Hu J. Shen L. Sun G. (2018). “Squeeze-and-excitation networks,” in Proceedings of the IEEE Conference on Computer Vision and Pattern Recognition (CVPR). (Lake city, UT, USA: IEEE), 7132–7141. doi: 10.1109/CVPR.2018.00745

[B16] Ioffe S. Szegedy C. (2015). “Batch normalization: accelerating deep network training by reducing internal covariate shift,” in International Conference on Machine Learning. (Lille, France: PMLR), 448–456. doi: 10.48550/arXiv.1502.03167

[B17] Jana S. Pareskh R. (2017). “Shape-based fruit recognition and classification,” in Proceedings of International Conference on Computational Intelligence, Communications, and Business Analytic (CIBA). (Kolkata, India: Springer). doi: 10.1007/978-981-10-6430-2_15

[B18] Jiao Y. Luo R. Li Q. Deng X. Yin X. Ruan C.. (2020). Detection and localization of overlapped fruits application in an apple harvesting robot. Electronics 9, 1023. doi: 10.3390/electronics9061023

[B19] Jocher G. Stoken A. Borovec J. Changyu L. Hogan A. Diaconu L.. (2020). ultralytics/yolov5: v3. 0. Zenodo.

[B20] Kurtulmus F. Lee W. S. Vardar A. (2011). Green citrus detection using “eigenfruit” color and circular Gabor texture features under natural outdoor conditions. Comput. Electr. Agric. 78, 140–149. doi: 10.1016/compag.2011.07.001

[B21] Lin T. Y. Dollár P. Girshick R. He K. Hariharan B. Belongie S. (2017). “Feature pyramid networks for object detection,” in Proceedings of the IEEE Conference on Computer Vision and Pattern Recognition (CVPR). (Honolulu, HI, USA: IEEE), 2117–2125. doi: 10.1109/CVPR.2017.106

[B22] Liu G. Mao S. Kim J. H. (2019). A mature-tomato detection algorithm using machine learning and color analysis. Sensors 19, 2023. doi: 10.3390/s19092023 31052169 PMC6539546

[B23] Liu G. Nouaze J. C. Touko M. P. L. Kim J. H. (2020). YOLO-Tomato: a robust algorithm for tomato detection based on YOLOv3. Sensors 20, 2145. doi: 10.3390/s20072145 32290173 PMC7180616

[B24] Liu S. Qi L. Qin H. Shi J. Jia J. (2018). “Path aggregation network for instance segmentation,” in Proceedings of the IEEE Conference on Computer Vision and Pattern Recognition (CVPR). (Lake City, UT, USA: IEEE), 8759–8768. doi: 10.48550/arXiv.1803.01534

[B25] Mbouembe P. L. T. Liu G. Sikati J. Kim S. C. Kim J. H. (2023). An efficient tomato-detection method based on improved YOLOv4-tiny model in complex environment. Front. Plant Sci. 14. doi: 10.3389/fpls.2023.1150958 PMC10106724 37077640

[B26] Padilla R. Netto S. L. Da Silva E. A. (2020). “A survey on performance metrics for object detection algorithms,” in International Conference on Systems, Signals, and Image Processing (IWSSIP). (Niteroi, Brazil: IEEE), 237–242. doi: 10.1109/IWWSSIP48289.2020.9145130

[B27] Payne A. Walsh K. Subedi P. Jarvis D. (2014). Estimating mango crop yield analysis using fruit at ‘stone hardening’ stage and night time imaging. Comput. Electr. Agric. 100, 160–167. doi: 10.1016/j.compag.213.11.011

[B28] Peixoto J. V. M. Neto C. M. Campos L. F. Dourado W. D. S. Nogueira A. P. Nascimento A. D. (2017). Industrial tomato lines: morphological properties and productivity. Genet. Mol. Res. 16, 1–15. doi: 10.4238/gmr16029540 28407184

[B29] Rahnemoofar M. Sheppard C. (2017). Deep count: Fruit counting based on deep simulated learning. Sensors 17, 905. doi: 10.3390/s17040905 28425947 PMC5426829

[B30] Rakun J. Stajnko D. Zazula D. (2011). Detection fruits in natural scenes using spatial-frequency based texture analysis and multi-view geometry. Comput. Electr. Agric. 76, 80–88. doi: 10.1016/j.compag.2011.01.007

[B31] Redmon J. Farhadi A. (2017). “Yolo9000: better, faster, stronger,” in Proceedings of the IEEE Conference on Computer Vision and Pattern Recognition. (Honolulu, HI, USA: IEEE), 7263–7271. doi: 10.1109/CVPR.2017.690

[B32] Redmon J. Farhadi A. (2018). Yolov3: An incremental improvement. arXiv preprint arXiv:1804.02767. doi: 10.48550/arXiv.1804.02767

[B33] Redmon J. Farhadi A. Divvala S. Girshick R. (2016). You only look once: unified, real-time object detection. In Proceedings of the IEEE Conference on Computer Vision and Pattern Recognition. (Las Vegas, NV, USA: IEEE), 779–788. doi: 10.48550/arXiv.1506.0240

[B34] Ren S. He K. Girshick R. Sun J. (2015). Faster r-cnn: towards real-time object detection with region proposal networks. In Proceedings of the International Conference on Neural Information Processing Systems 39. (Montreal, QC, Canada: IEEE) 91–99. doi: 10.1109/TPAMI.2016.2577031

[B35] Santo T. T. de Souza L. L. dos Santos A. A. Avila S. (2020). Grape detection, segmentation, and tracking using deep neural networks and three-dimensional association. Comput. Elects. Agric. 170, 105247. doi: 10.1016/compag.2020.105247

[B36] Szegedy C. Ioffe S. Vanhoucke V. Alemi A. A. (2017). “Inception-v4, Inception-Resnet and the impact of residual connections on learning,” in Proceedings of the Thirty-first AAAI Conference on Artificial Intelligence (San Francisco, CA, USA: AAAI Press). doi: 10.1609/aaai.v31i1.11231

[B37] Tan M. Pang R. Le Q. V. (2020). “Efficientdet: scalable and efficient object detection,” in Proceedings of the IEEE/CVF Conference on Computer Vision and Pattern Recognition (CVPR). (WA, USA: IEEE), 10781–10790. doi: 10.1109/CVPR42600.2020.01079

[B38] Wang C. Y. Boschkovskiy A. Liao H. Y. M. (2022). “Yolov7: trainable bag-of-freebies sets new state-of-the-art for real-time object detectors,” in Proceedings of the IEEE/CVF Conference on Computer Vision and Pattern Recognition (CVPR). (Vancouver, CANADA: IEEE), 7464–7475. doi: 10.48550/arXiv.2207.02696

[B39] Wang C. Y. Liao H. Y. M. Wu Y. H. Chen P. Y. Hsieh J. W. Yeh I. H. (2020a). “CSPNet: a new backbone that can enhance learning capability of CNN,” in Proceedings of the IEEE/CVF Conference on Computer Vision and Pattern Recognition Workshops (CVPR). (Seattle, WA, USA: IEEE), 390–391. doi: 10.1109/CVPRW50498.2020.00203

[B40] Wang J. Chen K. Xu R. Liu Z. Loy C. C. Lin D. (2019). “CARAFE: Content-aware reassembly of features,” in Proceedings of the IEEE/CVF International Conference on Computer Vision (ICCV). (Seoul, Korea: IEEE), 3007–3016. doi: 10.48550/arXiv.1905.02188

[B41] Wang X. Liu J. Liu G. (2021). Diseases detection of occlusion and overlapping tomato leaves based on deep learning. Front. Plant Sci. 12, 792244. doi: 10.3309/fpls.2021.792244 34956290 PMC8702556

[B42] Wang Q. Wu B. Zhu P. Li P. Zuo W. Hu Q. (2020b). “ECA-Net: efficient channel attention for deep convolutional neural networks,” in Proceedings of the IEEE/CVF Conference on Computer Vision and Pattern Recognition. 11534–11542. doi: 10.1109/CVPR42600.2020.01155

[B43] Woo S. Park J. Lee J. Y. Kweon I. S. (2018). “Cbam: convolutional block attention module,” in Proceedings of the European Conference on Computer Vision (ECCV). (Munich, Germany: Spinger), 3–19. doi: 10.1007/978-3-030-01234-2_1

[B44] Yang Q. Chen C. Dai J. Xun Y. Bao G. (2020). Tracking and recognition algorithm for robot harvesting oscillating apples. Int. J. Agric. Biol. Eng. 13, 163–170. doi: 10.25165/j.ijabe.20201305.5520

[B45] Zhang H. Chang H. Ma B. Wang N. Chen X. (2020). “Dynamic r-cnn: towards high quality object detection via dynamic training,” in European Conference on Computer Vision (ECCV). (Glasgow, UK: Springer, Cham, Computer Vision-ECCV), 260–275. doi: 10.1007/978-3-030-58555-6_16

[B46] Zhao Y. Gong L. Zhou B. Huang Y. Liu C. (2016b). Detecting tomatoes in greenhouse scenes by combining AdaBoost classifier and color analysis. Biosyst. Eng. 148, 127–137. doi: 10.1016/j.biosystemseng.2016.05.001

[B47] Zhao C. Lee W. S. He D. (2016a). Immature green citrus detection based on color feature and sum of absolute transformed difference (SATD) using color images in the citrus grove. Comput. Electr. Agric. 124, 243–253. doi: 10.1016/j.compag.2016.04.009

